# From Single-Chain Polymeric Nanoparticles to Interpenetrating Polymer Network Organogels: A One-Pot Fabrication Approach

**DOI:** 10.3390/gels11020122

**Published:** 2025-02-07

**Authors:** Selin Daglar, Demet Karaca Balta, Binnur Aydogan Temel, Gokhan Temel

**Affiliations:** 1 Department of Chemistry, Yildiz Technical University, Davutpasa Campus, Istanbul 34210, Turkey; selin.daglar@sabanciuniv.edu; 2 Sabanci University Nanotechnology Research and Application Center (SUNUM), Istanbul 34956, Turkey; 3 Department of Pharmaceutical Chemistry, Faculty of Pharmacy, Bezmialem Vakif University, Fatih, Istanbul 34093, Turkey; baydogan@bezmialem.edu.tr; 4 Department of Polymer Material Engineering, Faculty of Engineering, Yalova University, Yalova 77200, Turkey; gtemel@yalova.edu.tr

**Keywords:** single-chain polymeric nanoparticles, chain folding, interpenetrating polymer network, organogel

## Abstract

In this study, we developed a novel one-pot synthesis method to fabricate well-defined single-chain polymeric nanoparticles (SCNPs) integrated with interpenetrating polymer network (IPN) systems. The synthesis process involved an initial intramolecular crosslinking of poly(methyl methacrylate-*co*-glycidyl methacrylate) to form SCNP followed by intermolecular crosslinking to produce single-chain nanogel (SCNG) structures. In addition, the achieved single-chain polymeric nanoparticle was subsequently incorporated into an IPN structure through urethane bond formation and a Diels–Alder click reaction involving furfuryl methacrylate (FMA) and bismaleimide (BMI). The thermal properties, swelling behaviors, and morphologies of the resulting SCNP-IPN systems were investigated. This work presents a novel strategy that integrates the single-chain folding concept with IPN systems, providing a promising platform for the development of robust and functional polymeric materials with potential applications in advanced materials science.

## 1. Introduction

The synthesis of polymeric nanoparticles has traditionally relied on methods such as emulsion polymerization, interfacial polymerization, self-assembly, solvent evaporation, and supercritical fluid technology. However, controlling nanoparticle size and tailoring their functional groups with precision remain significant challenges in these conventional approaches [1,2]. In recent years, advances in the field of single-chain polymeric nanoparticles (SCNPs) have garnered substantial attention due to their unique properties and diverse applications.

SCNPs are a distinct class of polymeric nanoparticles formed through the intramolecular folding or collapse of individual polymer chains. These nanoscale structures, with dimensions ranging from 1.5 to 20 nm, exhibit controlled morphologies and surface functionalities, enabling their use in drug delivery systems, catalysis, sensors, dye-release applications, nanoreactors, and targeted therapies [3,4,5,6,7,8,9,10,11]. The ability to precisely control size and functionality during SCNP synthesis makes them attractive as analogues or mimics of protein-based materials. Furthermore, intramolecular chain folding enhances their interaction with target molecules by increasing surface area and optimizing structural accessibility [12,13,14].

The synthesis of SCNPs is highly dependent on the concentration of precursor polymer chains in the reaction medium. At ultra-dilute concentrations (below 1 mg/mL), intramolecular crosslinking dominates, whereas intermolecular crosslinking and network formation become prominent at higher concentrations. SCNPs are typically synthesized in ultra-dilute solutions to minimize interchain interactions, resulting in compact and homogeneous structures [12,13].

Gels, in general, are three-dimensional networks of crosslinked polymers or colloidal particles that can trap liquids or semi-solids within their structure, giving them a unique combination of solid-like and liquid-like properties [15,16]. Among gel systems, interpenetrating polymer networks (IPNs) have emerged as a versatile subclass due to their biocompatibility, mechanical strength, and adaptability to diverse functionalities. IPNs are defined as a combination of two or more polymer networks that coexist without covalent bonds. In the case of full IPNs, both networks are independently crosslinked, offering improved mechanical and thermal durability as well as enhanced functionality [17,18,19].

In this study, we report the development of a sequential synthesis strategy to integrate SCNPs with full-IPN systems, addressing the need for robust and multifunctional polymeric materials. The SCNP precursor was synthesized by intramolecularly crosslinking poly(methyl methacrylate-*co*-glycidyl methacrylate) (PMGA) copolymers through a reaction between the epoxy groups of glycidyl methacrylate and thiol groups of 2,2′-(ethylenedioxy)diethanethiol (DODT) in an ultra-dilute medium. The resulting SCNP was then incorporated into a full-IPN structure through a one-pot synthesis involving methyl methacrylate (MMA), furfuryl methacrylate (FMA), bismaleimide (BMI), and triethylamine (TEA) in DMF at 80 °C.

The SCNP-IPN network formation involved two complementary crosslinking mechanisms: urethane bond formation between hydroxyl (-OH) groups on the SCNP and isocyanate (NCO) groups of Ongronat, and a Diels–Alder click reaction between the furan ring in FMA and the maleimide structure in BMI. This synergistic approach allowed for the simultaneous creation of a homogeneously interpenetrated SCNP-IPN system with enhanced thermal properties.

By combining SCNP synthesis and organogel formation into a single system, this study demonstrates a transition from intramolecular crosslinking (SCNP) to single-chain nanogels (SCNGs), culminating in a robust SCNP-IPN network. The integration of furan and BMI functionalities within the same reaction medium highlights a novel method for developing advanced polymeric materials.

## 2. Results and Discussion

**Scheme 1:**
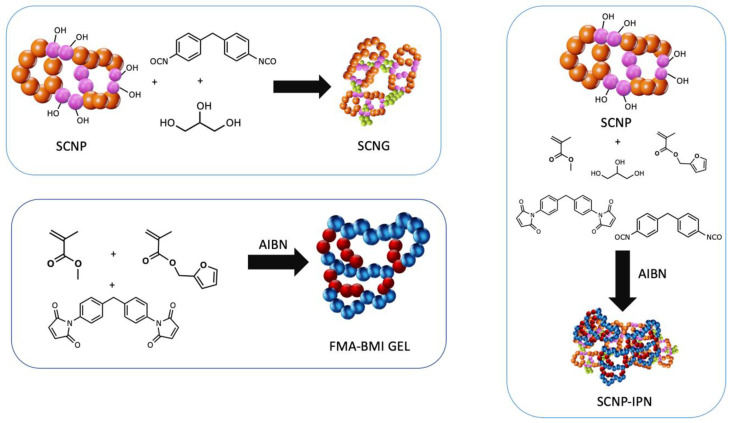
Overall mechanism of the syntheses of SCNG, FMA-BMI, and SCNP-IPN gels.

This study aimed to integrate SCNP systems into an interpenetrating polymer network (IPN) structure. In order to achieve corresponding incorporation, multicomponent organogels were prepared via one-pot process in the presence of hydroxy functional single-chain polymer nanoparticles (SCNPs), furfuryl methacrylate (FMA), bismaleimide (BMI), methyl methacrylate, and glycerol. Initially, the formation of SCNP was carried out by intramolecular crosslinking of epoxy functionalized linear copolymer chains and di-functional thiol crosslinker. SCNP-IPNs were subsequently prepared by combining resulting SCNP with external crosslinkers under radical initiation to create hybrid interpenetrating polymer networks with enhanced structural properties. Finally, for comparison, SCNG was synthesized by crosslinking SCNP nanoparticles in the presence of functional crosslinkers, and FMA-BMI gels were created through the polymerization of methacrylates with a bismaleimide and a radical initiator (see Scheme 1).

### 2.1. Synthesis and Characterization of SCNP

**Scheme 2:**
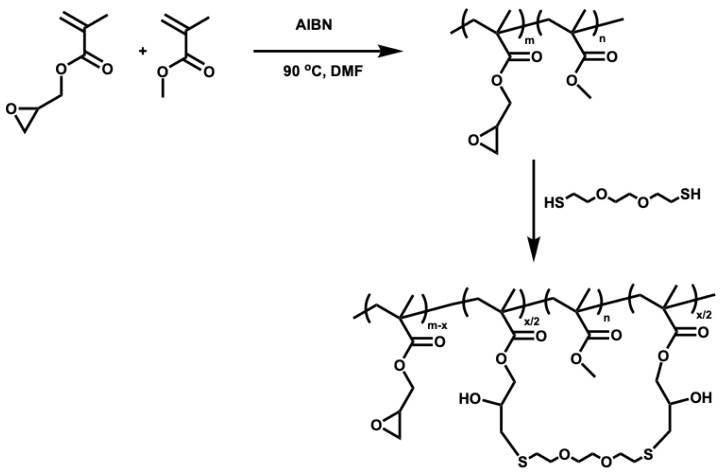
Synthesis of poly(methyl methacrylate-*co*-glycidyl methacrylate) (PMGA) and SCNP formation.

**Figure 1:**
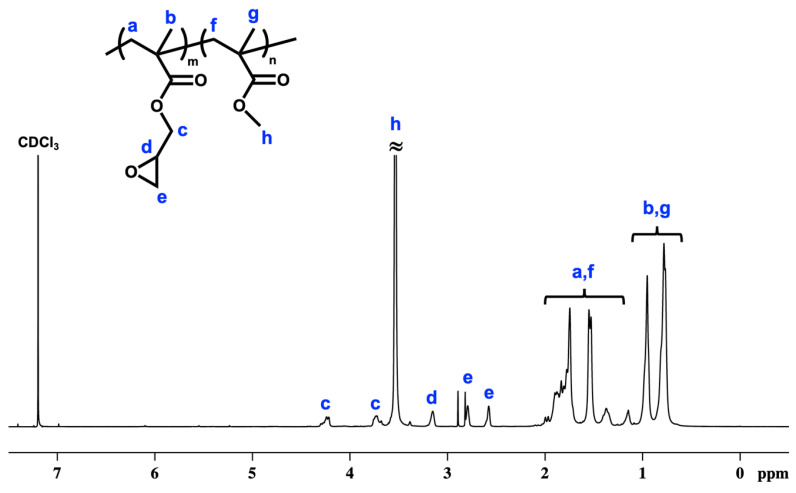
^1^H-NMR of poly(methyl methacrylate-*co*-glycidyl methacrylate) (PMGA) in CDCl_3_.

The copolymerization of MMA and GMA was carried out at 90 °C in a 90:10 feed ratio using classical free radical polymerization (Scheme 2). Successful preparation of the copolymer was confirmed by ^1^H-NMR (Figure 1) and GPC (Table 1).

The two similar peaks with approximately the same intensity that appeared at 3.8 and 4.31 ppm (c) are related to the geminal protons of the methylene group adjacent to the ester oxygen. The protons of the epoxide ring (d and e) were observed at 3.24 ppm, 2.8 ppm and 2.64 ppm. The single peak that appeared at 3.6 ppm (h) is related to the –OCH_3_ protons in the MMA segment. The methylene protons of the copolymer main chains appeared between 0.5 and 2.0 ppm (a, b, f, g) (Figure 1). According to ^1^H-NMR results, copolymer composition was found to be [MMA]:[GMA] = 88:12. The average molecular weight (*M*_n_) of PMGA was found to be 55,600 g mol^−1^ using GPC. The *M*_n_ value was also used to estimate the number of GMA and MMA units as approximately 65 and 462, respectively [20,21].

2,2′-(Ethylenedioxy)diethanethiol (DODT) was used as a crosslinker with the achieved copolymer PMGA in order to obtain an intramolecularly crosslinked nanoparticle in a dilute medium (c = 0.3 mg mL^−1^) (Scheme 2). The transformation of PMGA into single-chain polymeric nanoparticles (SCNPs) through intramolecular crosslinking resulted in significant changes in molecular and thermal properties, highlighting the structural compaction achieved during synthesis. The *M*_n_ of the SCNP decreased markedly from 55,600 g mol^−1^ to 32,300 g mol^−1^, accompanied by a slight reduction in dispersity (*Đ*) from 2.9 to 2.6, indicating enhanced uniformity (Table 1). Dynamic light scattering (DLS) measurements showed a reduction in PDI from 0.481 for the linear polymer to 0.366 for the SCNP. This decrease indicates that the intramolecular cross-linking process enhanced size uniformity by reducing intermolecular interactions and aggregation, resulting in compact, globular nanoparticles. The moderate polydispersity of the SCNP reflects the inherent molecular weight dispersity of the linear precursor but also demonstrates the effectiveness of the folding process [22].

Additionally, differential scanning calorimetry (DSC) showed an increase in glass transition temperature (T_g_) from 84.62 °C to 96.95 °C (Table 1), reflecting the restricted segmental motion and enhanced rigidity of the SCNPs [23].

**Figure 2:**
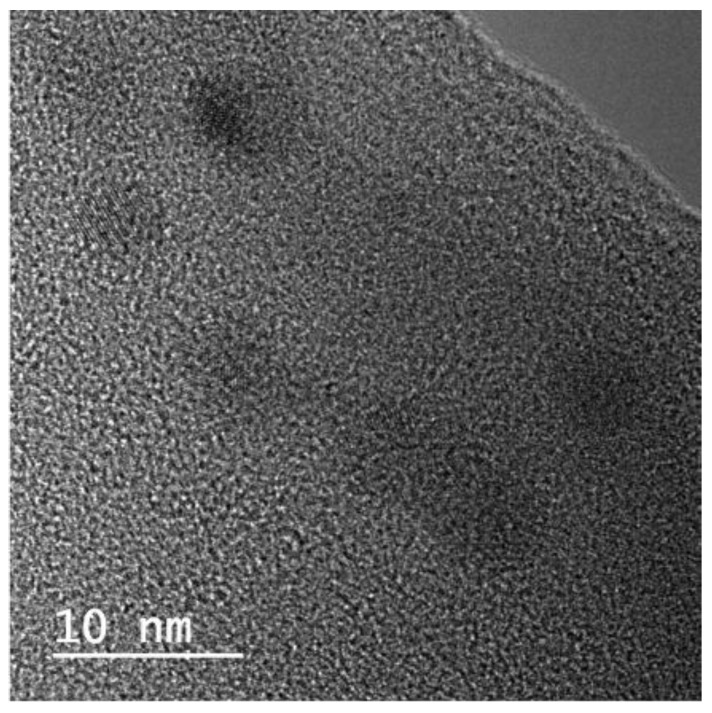
TEM image of SCNP.

The TEM image provides direct evidence of SCNP formation, confirming the successful intramolecular crosslinking of the polymer chains. Figure 2 shows a representative TEM image of SCNPs, displaying compact, globular structures with sizes in the range of 10–20 nm, confirming intramolecular crosslinking and efficient chain collapse. The observed nanoscale features, with a uniform and dense distribution, reflect the compact structure characteristic of SCNPs. The absence of significant aggregation suggests that the crosslinking process effectively suppressed intermolecular interactions, leading to well-defined individual nanoparticles. This observation is in good accordance with the hydrodynamic diameter of 11.10 nm obtained through dynamic light scattering (DLS), further supporting the successful synthesis of compact SCNPs [5,23].

### 2.2. Preparation and Characterization of SCNP-IPN Gels

SCNPs were successfully integrated into the IPN structure through the formation of urethane bonds and a Diels–Alder click reaction involving furfuryl methacrylate (FMA) and bismaleimide (BMI), as depicted in Scheme 1.

**Scheme 3:**
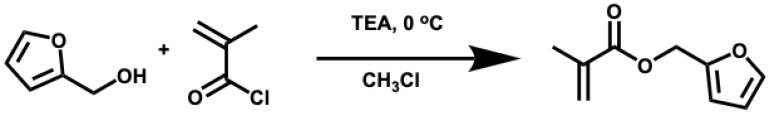
Synthesis of furfuryl methacrylate (FMA).

**Figure 3:**
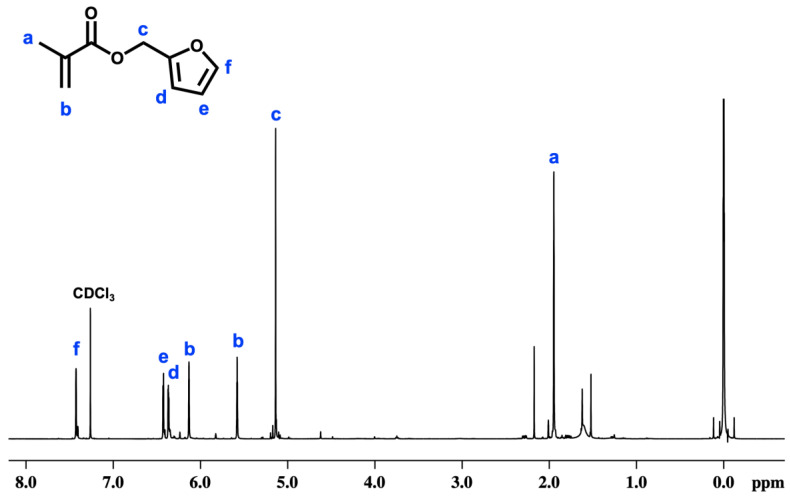
^1^H-NMR of furfuryl methacrylate (FMA) in CDCl_3_.

Initially, FMA was synthesized via a straightforward esterification reaction using furfuryl alcohol and methacryloyl chloride, as illustrated in Scheme 3. The successful synthesis of FMA was confirmed by ^1^H-NMR analysis (Figure 3), which revealed all the characteristic peaks corresponding to its chemical structure, including signals for the furfuryl (c–f) and methacrylate (a and b) groups. The observed proton peaks were in full agreement with the expected values, confirming the compound’s purity and composition [24].

To enable a comprehensive characterization of the one-pot IPN gels, two additional gel systems, FMA-BMI and SCNG, were independently synthesized. These systems served as reference materials, allowing for a more detailed comparative analysis of the structural, mechanical, and functional properties of the IPN gels. The FMA-BMI gel was prepared using furfuryl methacrylate (FMA) and bismaleimide (BMI) via a Diels–Alder reaction, while the SCNG gel was synthesized to isolate the contributions of single-chain polymeric nanoparticles (SCNPs) to the final IPN architecture. This comparative approach provided valuable insights into the individual and combined roles of these components within the IPN structure.

**Figure 4:**
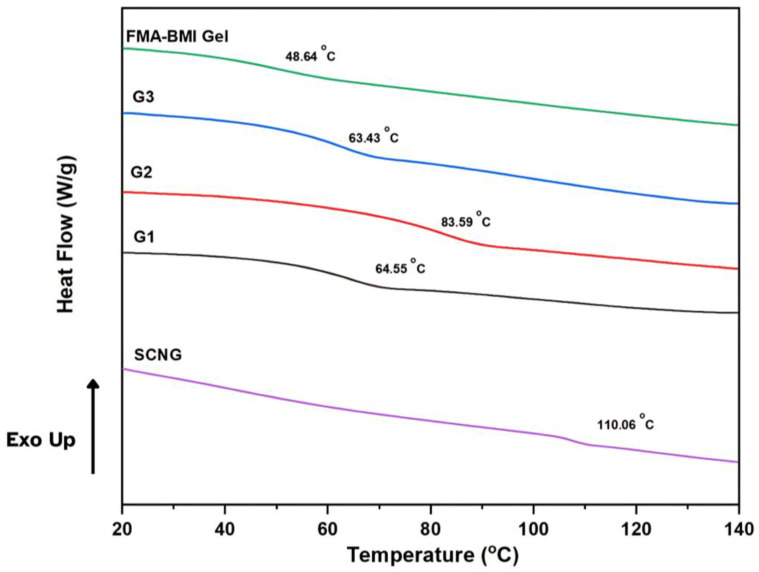
DSC thermograms (second heating scan) of gel products.

The initial characterization of the one-pot IPN gels was performed using differential scanning calorimetry (DSC) (Figure 4). The DSC thermograms demonstrated that each material exhibited a distinct glass-transition behavior within the temperature range of approximately 40 °C to 120 °C. The SCNG sample demonstrated the highest glass transition temperature (T_g_) at around 110 °C, which could be attributed to its comparatively more rigid or highly crosslinked network structure. In contrast, the FMA–BMI gel exhibited the lowest transition temperature, approximately 49 °C, indicating a less rigid and more flexible polymer network. The interpenetrating polymer networks (IPNs), denoted as G1, G2, and G3, exhibited T_g_ values that decreased between these two extremes, ranging from approximately 64 °C to 84 °C. Among these, G2 displayed the highest T_g_ (∼84 °C), suggesting that this formulation achieved a more effective or denser crosslinking compared to G1 and G3. In contrast, G1 and G3 exhibited lower T_g_ values (both ∼64 °C), indicating a less rigid network and a different balance between flexibility and rigidity.

Overall, the DSC data exhibited the significant impact of compositional variations—such as the ratio of isocyanates, glycerol, FMA, and BMI—on the thermal properties of the materials.

**Figure 5:**
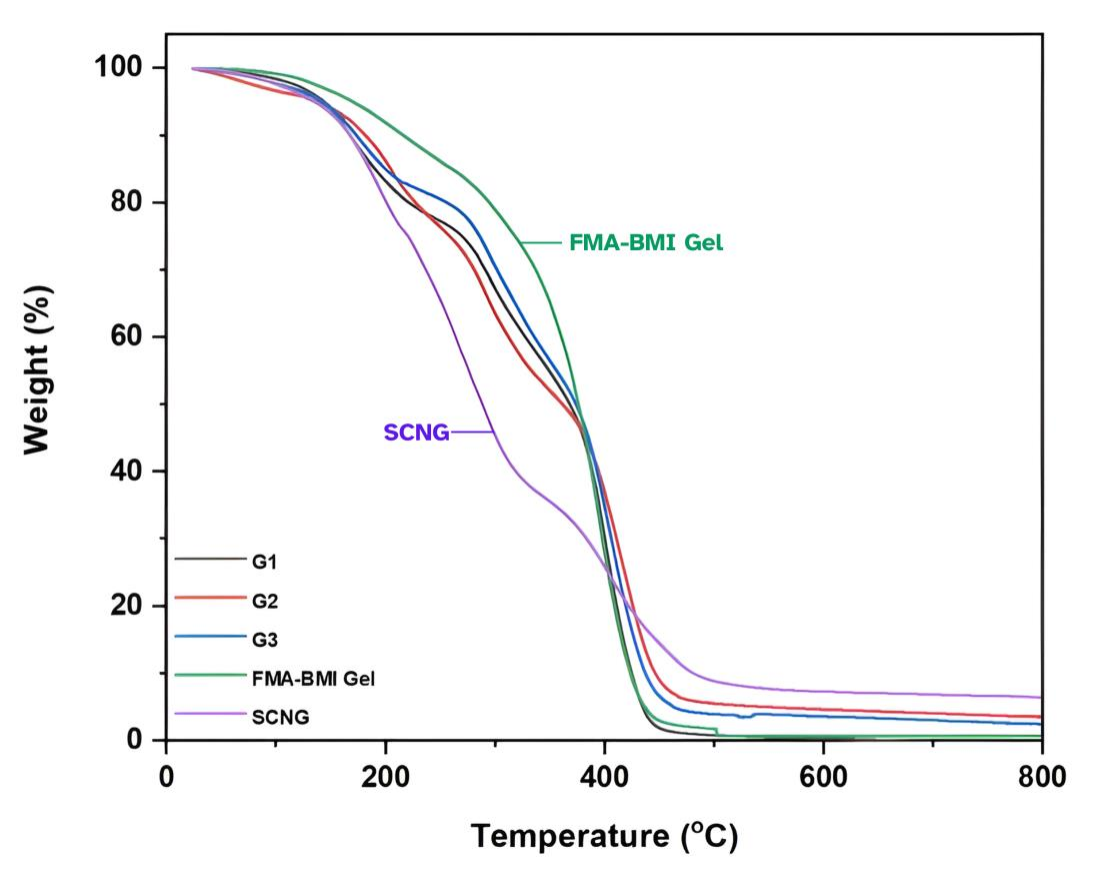
TGA curves of G1, G2, G3, FMA-BMI gel, and SCNG under nitrogen atmosphere.

According to the TGA results, there were clear differences in the thermal decomposition behavior of the FMA–BMI gel compared with the SCNG (Figure 5). The FMA–BMI gel showed a prominent weight-loss behavior between roughly 200 °C and 400 °C, rapidly degrading to very low residual mass above 500 °C. In contrast, the SCNG sample showed a more gradual mass-loss profile, with its main decomposition shifted to higher temperatures and a larger char yield at elevated temperatures. These observations suggest that the SCNG’s architecture, with additional intrachain or interchain crosslinks, enhances thermal stability by limiting chain movement and delaying the onset of extensive bond scission. Although the FMA–BMI gel is initially stable, it loses most of its mass in a narrower temperature interval, indicating that its network degrades more readily once key covalent bonds begin to break. However, IPN structures are found within the spectrum of these two extremes. Overall, these results demonstrate the effect of crosslink density, backbone structure, and network design during the thermal degradation pathways of the resulting polymeric products [25].

**Figure 6:**
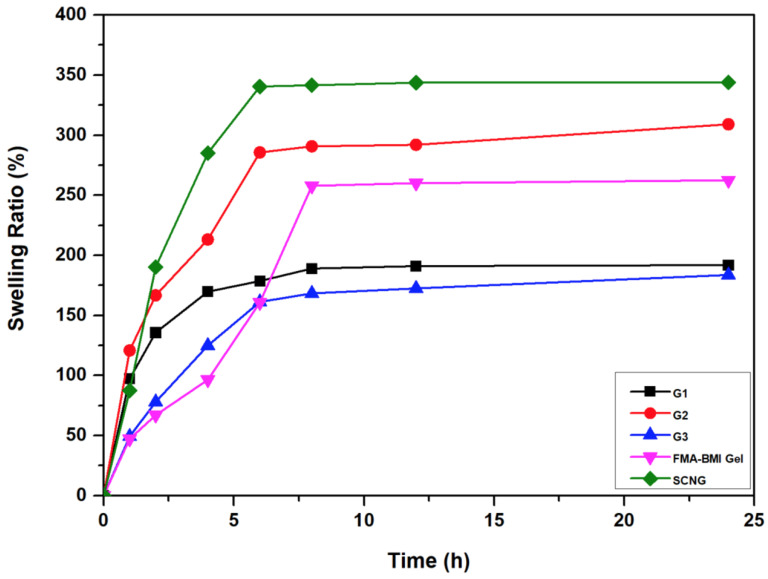
Swelling degree with time of gel samples.

A critical observation from the swelling-versus-time data in DMF is the significant influence of network architecture and crosslink density on the gel’s solvent uptake [26]. Despite DMF being a polar organic solvent, the distinct swelling behaviors observed can be attributed to variations in crosslink density, crosslinker functionality, and polymer-solvent compatibility. Although it is considered that the SCNG sample has the highest crosslinking degree according to the DSC results, it exhibited rapid swelling behavior, exceeding 350% within a few hours, indicative of an open network structure with high compatibility for DMF solvent (Figure 6). In contrast, the G-series gels (G1, G2, and G3) showed a clear reduction in swelling capacities. This decrease correlates with compositional differences, particularly the SCNP content and the types of isocyanate crosslinkers (Ongronat and BMI) incorporated. G3 (blue triangles), with the lowest swelling, likely has a denser network or a composition less favorable for DMF diffusion (Figure 6). G1 (black squares) and G2 (red circles) exhibited intermediate swelling levels, reflecting how even modest changes in crosslinker type, ratio, or the inclusion of SCNPs can significantly influence network architecture and solvent uptake. These findings highlight the critical role of polymer formulation parameters—such as SCNP loading, crosslinker content, and functional monomer selection—in shaping network morphology and solvent absorption properties. Ultimately, these results provide a framework for engineering polymer networks with tailored solvent absorption characteristics through the precise control of formulation and structure.

**Figure 7:**
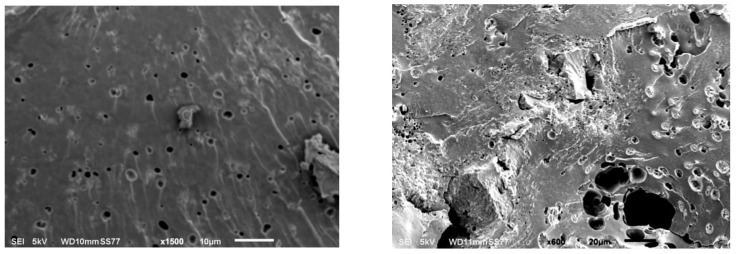
SEM images of G2 (**left**, scale bar: 10 μm) and G3 (**right**, scale bar: 20 μm).

From a morphological perspective, G2 exhibited a relatively dense matrix with small, sporadic pores, suggesting a more uniform network structure (Figure 7). In contrast, G3 displayed a heterogeneous topography characterized by larger pores and a more pronounced porous architecture. These differences are likely due to variations in SCNP content. The extensive porosity and larger cavities observed in G3 may result from higher shrinkage or less uniform polymerization, whereas the smoother and denser regions in G2 indicate a more homogeneous network formation. These SEM images demonstrated that changes in composition or synthesis parameters could significantly influence the microstructure of the gels.

## 3. Conclusions

In this study, SCNP-based materials, including SCNG, FMI-BMI gel, and SCNP-IPNs (G1, G2, and G3), were successfully synthesized and characterized. The swelling ratio analysis revealed that SCNG exhibited the highest swelling capacity, attributed to its soft and flexible structure, whereas FMI-BMI gels showed more restricted swelling due to their densely cross-linked networks or low solvent interaction. Thermal analyses using DSC and TGA indicated that SCNG demonstrated superior thermal resistance compared to the FMI-BMI gels, likely resulting from the efficient incorporation of isocyanate groups within the SCNP framework. The variations in gel formulations (G1, G2, and G3) highlighted that SCNP and crosslinker concentrations played a significant role in modulating both swelling behavior and thermal properties. Importantly, the novelty of this study lies in the use of functional SCNPs as crosslinkers, coupled with a one-pot strategy that combines free radical and step-growth polymerizations, offering an innovative and efficient route to fabricate advanced polymeric networks. These findings underscore the potential of SCNP-based materials for a wide range of applications, including responsive hydrogels, advanced composite materials, and biomedical devices, where tunable swelling and thermal properties are essential.

## 4. Materials and Methods

### 4.1. Materials

Furfuryl alcohol (98%, Sigma-Aldrich, St. Louis, MO, USA), methacryloyl chloride (97%, contains ppm monomethyl ether hydroquinone as stabilizer, Sigma-Aldrich, St. Louis, MO, USA), glycerol (≥99.5%, Sigma-Aldrich, St. Louis, MO, USA), triethyl amine (TEA, ≥99%, Sigma-Aldrich), potassium hydroxide (KOH, Supelco, St. Louis, MO, USA), and sodium bicarbonate (NaHCO₃, ≥99.5%, Sigma-Aldrich, St. Louis, MO, USA) were used as received. 2,2′-Azobis(2-methylpropionitrile) (AIBN, Aldrich, St. Louis, MO, USA) was purified by recrystallization from ethanol. Methyl methacrylate (Merck, Darmstadt, Germany) and glycidyl methacrylate (97%, contains 100 ppm monomethyl ether hydroquinone as inhibitor, Sigma-Aldrich, St. Louis, MO, USA) were de-inhibited by passing over basic alumina. The Ongronat^®^ 3600 monomeric MDI isomer mixture (BorsodChem, Kazincbarcika, Hungary, 2,4′-MDI and 4,4′-MDI in a nominal ratio of 50:50) and 2,2′-(ethylenedioxy)diethanethiol (DODT, 95%, Sigma-Aldrich, St. Louis, MO, USA) were used as received. *N*,*N*-Dimethyl formamide (DMF), acetone, methanol, tetrahydrofuran (THF), chloroform (CHCI_3_), and n-hexane were purchased from Sigma-Aldrich, St. Louis, MO, USA and used without further purification.

### 4.2. Instruments

^1^H-NMR spectra were recorded on a Bruker Avance Spectrometer (500 MHz, Karlsruhe, Germany) using CDCI_3_ as a solvent. FT-IR analyses were performed on Nicolet IS10 (Thermo Electron Scientific Instruments LLC, Madison, WI, USA) equipped with an ATR accessory and a diamond crystal. The spectra were recorded in the infrared range of 4000–500 cm^−1^. The molecular weight of the polymer was determined by a gel permation chromotography (GPC) instrument (Malvern Panalytical, Worcestershire, UK), Viscotek GPCmax Autosampler system, consisting of a pump, three ViscoGEL GPC columns (G2000HHR, G3000HHR, and G4000HHR), and a Viscotek differential refractive index detector. Tetrahydrofuran was used as the eluent at a flow rate of 1.0 mL min^−1^. The average molecular weights were determined using linear polystyrene standards. The data were evaluated using Viscotek OmniSEC Omnie01 software (Malvern Panalytical, Worcestershire, UK). The average particle size and particle size distributions of the polymeric nanoparticles were determined using a Malvern NanoZSP dynamic light scattering (DLS) instrument (Worcestershire, UK) in acetone at 25 °C with a measurement angle of 173° (backscattering). Mean values of the number-weighted diameter were calculated from five measurements in each case. Differential scanning calorimetry (DSC) measurements were performed on TA Instruments Discovery DSC 250 (New Castle, DE, USA) with a heating rate of 10 °C min^−1^ under a nitrogen atmosphere. For Tg determination, three cycles from 25 °C to 150 °C and subsequent cooling to 25 °C were measured. The Tg was defined as the turning points of the stages in the second heating curve. Thermogravimetric analyses were recorded on a Shimadzu DTG 60 (Kyoto, Japan) with a heating rate of 10 °C min^−1^ under a nitrogen atmosphere. Scanning electron microscope analyses were performed with JEOL JSM 6010 W-SEM (Tokyo, Japan) (equipped with EDS and EBSD Systems). The gel samples were dried once again under vacuum at 50 °C prior to analysis. The samples were coated with Au/Pd sputter and then analyzed on a JEOL JSM 6010 W-SEM (equipped with EDS and EBSD Systems). HR-TEM measurement was performed using a JEOL JEM-ARM200CFEG UHR-TEM (Tokyo, Japan) (equipped with STEM, Cs corrected STEM, EDS, Gatan Quantum GIF and Digital CCD Camera). The SCNP sample was prepared by drop coating a dilute solution of the sample (in acetone c = 0.01 mg mL^− 1^) on a holey carbon-coated copper grid.

### 4.3. Synthesis of Poly(methyl methacrylate-co-glycidyl methacrylate) (PMGA)

The copolymer was synthesized via free radical polymerization using glycidyl methacrylate (0.28 mL, 2.1 mmol) and methyl methacrylate (2 mL, 19 mmol) at a 90:10 molar ratio (MMA:GMA). 2,2′-Azobis(2-methylpropionitrile) (AIBN) (0.211 mmol, 0.0328 g) served as the initiator, and the reaction was conducted in 2 mL of dimethylformamide (DMF) at 90 °C for 5 h under a nitrogen atmosphere. After completion of the reaction, the resulting copolymer was purified by precipitation in excess methanol. The precipitated product was collected by filtration using filter paper and dried in a vacuum oven (yield: 79%) [21].

### 4.4. Formation of Single-Chain Polymeric Nanoparticles (SCNPs) from Poly(methyl methacrylate-co-glycidyl methacrylate)

Potassium hydroxide (KOH, 11 mg, 1.28 mmol) and 2,2′-(ethylenedioxy)diethanethiol (DODT, 19.5 µL, 0.1 mmol) were dissolved in acetone (800 mL) in an ice bath. While the solution was vigorously stirred under a nitrogen atmosphere, PMGA (300 mg) dissolved in acetone (100 mL) was added dropwise over 2 h. After the addition was complete, the reaction mixture was stirred for an additional 2 days at room temperature. At the end of this period, the solvent was completely evaporated and the resulting solid polymer was dissolved in chloroform. The chloroform solution was then washed three times with distilled water to neutralize the solution. The organic phase was dried over anhydrous sodium sulfate, filtered, and concentrated by evaporating the chloroform under reduced pressure. The residue was precipitated into cold hexane and dried in a vacuum oven to yield the final product (yield: 63%) [23].

### 4.5. Synthesis of Furfuryl Methacrylate (FMA)

Furfuryl alcohol (2 mL, 1 equivalent) and triethylamine (TEA, 3.3 mL, 1 equivalent) were dissolved in chloroform in a reaction flask and cooled in an ice bath. Methacryloyl chloride (2 mL, 1.1 equivalents) dissolved in chloroform was added dropwise to the mixture using a syringe, with continuous stirring. After the addition was complete, the reaction mixture was removed from the ice bath and stirred at room temperature for 24 h. Upon completion of the reaction, the mixture was extracted 3–4 times with a sodium bicarbonate (NaHCO_3_) solution to neutralize any residual acid. The organic phase was subsequently washed with water to ensure neutrality and cured product was purified by column (yield: 77%) [27].

### 4.6. Preparation of Single-Chain Nanogel (SCNG)

SCNP (50 mg), glycerol (110 mg), Ongronat 3600 (150 mg), and 0.5 mL of DMF were mixed in a tube. Gel formation was observed within 2 h under at 80 °C. The gel was dried in a vacuum oven at 50 °C for 2 days.

### 4.7. Preparation of FMA-BMI Gel

MMA (1.10 mL), FMA (50 mg), BMI (10 mg), AIBN (1 wt% based on the total FMA and MMA ratio), TEA (used as a catalyst), and 0.5 mL of DMF were mixed in a tube. Gel formation was observed within 2 h at 80 °C. The gel was dried in a vacuum oven at 50 °C for 2 days.

### 4.8. One-Pot SCNP-IPN Gel Preparation

Three series of gels with different weight ratios (G1: 1:1, G2: 1:0.5, and G3: 0.5:1) were prepared to form interpenetrating polymer network (IPN) gels (Table 2). AIBN was used as an initiator in a proportion of 1 wt% based on the total monomer (FMA and MMA) ratio. The preparation procedure involved combining the following components in a tube: SCNP, glycerol, Ongronat 3600, MMA, FMA, BMI, AIBN, TEA (used as a catalyst), and 0.5 mL of DMF. Gel formation was observed within 2 h at 80 °C (Table 2). After gel formation, the three series of gels were dried in a vacuum oven at 50 °C for 2 days [28,29]. The resulting gel products have a shiny orange color and semi-transparent appearance.

## Figures and Tables

**Table 1:** Molecular weight results (*M*n and *M*w), dispersity (*Đ*), glass transition temperature (T_g_), and particle size values of copolymer precursor and its SCNP.

Run	*M*_n_ ^a^ (Da)	*M*_w_ (Da)	*Đ* ^a^	T_g_^b^ (°C)	D_H_^c^ (nm)
PMGA	55,600	161,240	2.9	84.62	11.69
SCNP	32,300	83,980	2.6	96.95	11.10

^a^ Determined by SEC according to linear polystyrene standards. ^b^ Glass transition temperature determined by DSC. ^c^ Particle size determined by DLS; samples suspended in THF.

**Table 2:** Formulations of the gels.

Entry	SCNP (mg)	Ongronat 3600 (mg)	Glycerin (mg)	FMA (mg)	MMA (mL)	BMI (mg)
G1	50	150	110	50	1.10	10
G2	50	150	110	25	0.55	5
G3	25	75	55	50	1.10	10

## Data Availability

The original contributions presented in this study are included in the article. Further inquiries can be directed to the corresponding author.
